# Vitamin A- and D-Deficient Diets Disrupt Intestinal Antimicrobial Peptide Defense Involving Wnt and STAT5 Signaling Pathways in Mice

**DOI:** 10.3390/nu15020376

**Published:** 2023-01-11

**Authors:** Louisa Filipe Rosa, Patricia P. Petersen, Lisa F. Görtz, Iris Stolzer, Valentina Kaden-Volynets, Claudia Günther, Stephan C. Bischoff

**Affiliations:** 1 Institute of Nutritional Medicine, University of Hohenheim, Fruwirthstr. 12, 70599 Stuttgart, Germany; 2 Department of Medicine 1, Friedrich-Alexander-University Erlangen-Nürnberg, 91054 Erlangen, Germany; 3 Deutsches Zentrum Immuntherapie, Friedrich-Alexander University Erlangen-Nuermberg, 91054 Erlangen, Germany

**Keywords:** vitamin A, vitamin D, antimicrobial peptides, gut barrier, Wnt signaling pathway, Jak/STAT, MAPk

## Abstract

Vitamin A and D deficiencies are associated with immune modulatory effects and intestinal barrier impairment. However, the underlying mechanisms remain unclear. C57BL/6J mice were fed either a diet lacking in vitamin A (VAd), vitamin D (VDd) or a control diet (CD) for 12 weeks. Gut barrier function, antimicrobial peptide (AMP) defense and regulatory pathways were assessed. VAd mice compared to CD mice showed a reduced villus length in the ileum (*p* < 0.01) and decreased crypt depth in the colon (*p* < 0.05). In both VAd- and VDd-fed mice, ileal α-defensin 5 (*p* < 0.05/*p* < 0.0001 for VAd/VDd) and lysozyme protein levels (*p* < 0.001/*p* < 0.0001) were decreased. Moreover, mRNA expression of lysozyme (*p* < 0.05/*p* < 0.05) and total cryptdins (*p* < 0.001/*p* < 0.01) were reduced compared to controls. Furthermore, matrix metalloproteinase-7 (Mmp7) mRNA (*p* < 0.0001/*p* < 0.001) as well as components of the Wnt signaling pathway were decreased. VAd- and VDd-fed mice, compared to control mice, exhibited increased expression of pro-inflammatory markers and β-defensins in the colon. Organoid cell culture confirmed that vitamins A and D regulate AMP expression, likely through the Jak/STAT5 signaling pathway. In conclusion, our data show that vitamin A and D regulate intestinal antimicrobial peptide defense through Wnt and STAT5 signaling pathways.

## 1. Introduction

Vitamin A (VA) and vitamin D (VD) are essential fat-soluble micronutrients, playing a key role in multiple systemic functions including immune function [1,2] and the regulation of the gastrointestinal tract. For example, VA and VD have been associated with the differentiation of intestinal epithelial cells, as well as the homeostasis of the mucosal barrier [3,4]. VA and VD deficiencies continue to be a public health problem. While mammals cannot synthesize VA by themselves and its supply relies completely on diet [5], humans can utilize two forms of VD, vitamin D3 (cholecalciferol) from fish or synthesized by UV radiation in the skin, and vitamin D2 (ergocalciferol) from fungi and other nutrients after conversion to vitamin D3 [6]. However, VD synthesis in the skin varies depending on clothes, temperature and seasons [7].

Deficiency of VA or VD has been associated with gastrointestinal infections, severe diarrhea, and intestinal barrier dysfunction [3,4,8,9,10], which is in turn considered to be a key determinant in the pathogenesis of a variety of diseases such as inflammatory bowel disease (IBD) [9,11,12], irritable bowel syndrome (IBS) [13,14], and metabolic diseases, e.g., diabetes and nonalcoholic fatty liver disease (NAFLD) [15,16]. Consistent with this, the absence of VD receptor (VDR) increased the risk for these diseases [17]. In turn, the administration of VD has been shown to be protective against dextran sulfate sodium (DSS)-induced colitis [4]. Moreover, human studies revealed that patients with Crohn’s disease have a higher prevalence of VA deficiency [10] and a down-regulation of VDR genes in the small intestine [18].

An important defense strategy of the gut is the production of antimicrobial peptides (AMPs) by specialized Paneth cells at the base of the crypts of Lieberkühn [19,20]. Paneth cell antimicrobials act as a second line of defense by protecting against harmful bacteria. Thus, a compromised antimicrobial Paneth cell defense promotes bacterial translocation and gastrointestinal diseases [21,22]. Several studies have shown that VD induces the expression of cathelicidin antimicrobial peptide (CAMP) and human β-defensin 2 (HBD-2) gene [23,24], as the vitamin D response element (VDRE) is located in the promoter region of these genes [25]. Moreover, VD has been associated with an increased antimicrobial activity against pathogens through direct killing by cathelicidin (LL-37) and enhancing phagosome maturation [26]. Similarly, all-trans retinoic acid (RA) was shown to induce cathelicidin and porcine (PR-39) expression [27], whereas HBD-2 and -3 were shown to be inhibited by RA [28]. Concordantly, Paneth cell VDR knockout mice exhibited impaired AMP release and microbiota composition, and showed reduced resistance to Salmonella infections and DSS-induced colitis [18]. In comparison, higher VD levels in colitis ulcerosa patients were associated with increased serum cathelicidin and reduced inflammation [29].

In various studies, VA and VD were able to improve the generation of Paneth cell antimicrobials, while inflammatory processes were reduced. However, the underlying mechanisms supporting the regulatory function of VA and VD on the intestinal barrier, especially on the antimicrobial defense, are still poorly understood. In the present study, we aimed to gather new insights in the interplay between vitamin intake and intestinal barrier integrity in young female C57BL/6J mice. Therefore, we assessed the role of VA- or VD-deficient diets on markers of intestinal barrier functions including AMPs and intestinal permeability. Furthermore, we investigated regulatory mechanisms that might be involved in these effects.

## 2. Materials and Methods

### 2.1. Experimental Setup, Animals, and Diets

The experiment was carried out with 36 female C57BL/6J mice, as they were determined to be more susceptible to diet-induced disorders [21,30]. Mice aged 6–8 weeks were housed in a specific pathogen-free (SPF) barrier facility with a controlled environment (12-h light/dark cycles) accredited by the Association for Assessment and Accreditation for Laboratory Animal Care International. All experiments were approved by the local Animal Care and Use Committee (Regional Council Stuttgart 312/14 EM).

Mice were randomly divided into three groups (*n* = 12 per group), receiving either a control diet (CD, Ssniff ^®^ EF R/M control, vitamin A 15000 IE, vitamin D 1500 IE), a vitamin A-deficient diet (ssniff^®^ EF R/M vitamin-A-free, vitamin A < 120 IE) or a vitamin D-deficient diet (ssniff^®^ EF R/M vitamin-D-free, vitamin D < 5 IE) for 12 weeks, respectively. The detailed compositions of the administered diets are shown in Table 1. Autoclaved tap water was offered ad libitum. Food intake, fluid intake, and body weight were assessed on a weekly basis throughout intervention. After 12 weeks, barrier tests were performed and animals were anesthetized by intraperitoneal injection of ketamine–xylazine (100:16 mg/kg body weight). Blood was collected by portal vein puncture. Liver and gut tissue specimens were collected and stored at −80 °C as well as preserved in neutral-buffered formalin for histomorphological investigation.

### 2.2. Measurement of Intestinal Permeability

To carry out intestinal permeability test using polyethylene glycol 4000 (PEG4000) in the urine or fluorescein isothiocyanate–dextran (FITC-D4000) in the portal vein plasma, mice fasted for 6 h after the 12-week diet phase. The procedure was performed as previously described [31].

### 2.3. Morphological Quantification by HE Staining and Immunofluorescence Staining

For histological evaluation, 5 µm thick paraffin sections of gut and liver tissue were stained with hematoxylin/eosin (HE) as described [32]. For fluorescence microscopy, 3 µm thick paraffin sections of ileum and colon were prepared. Samples were deparaffinized in Roticlear (Roticlear^®^, Carl Roth GmbH & Co KG, Karlsruhe, Germany) and rehydrated in descending alcohol series (100% EtOH, 96% EtOH, 70% EtOH). For lysozyme staining, slides were pretreated with proteinase K. After short washing with PBS, sections were incubated with PBS containing 5% Normal Donkey Serum (NDS, 0.1% Triton X-100, 0.02% sodium acid) for 1 h at room temperature (RT). Primary polyclonal rabbit anti-DEFA5 (1:100 diluted in PBS; AB912Mu01, dianova GmbH, Hamburg, Germany) or anti-lysozyme antibody (1:100 diluted in PBS; A0099, Dako, Glostrup, Denmark) was applied overnight at 4 °C. Afterwards, samples were washed three times in PBS for 5 min each and incubated with donkey anti-rabbit Alexa Fluor 594-conjugated antibody diluted in PBS (1:800) (145043, Jackson ImmunoResearch Laboratories, West Grove, PA, USA) for 1 h at RT. Staining was followed by three washes of 5 min each with PBS and nuclei visualization was performed with 4′,6-diamidino-2-phenylindole (DAPI) (Hoechst 33258 dye, 5 µg/mL in PBS; Sigma-Aldrich, Darmstadt, Germany) for 5 min at RT. For microscopy examination, sections were mounted with Fluoromount-G (0100-01, Southernbiotech, Birmingham, AL, USA). Quantitative analysis was performed using optical density measurements by a fluorescence microscope (Axiovert 200 M, AxioVision 4.8.2 SP3; Carl Zeiss AG, Aalen, Germany). An automatic measurement program was used to determine the total area of the fluorescence signal (%) in a fixed frame (width: 200.273 µm; length: 333.78 µm). For each slide, 5 images were created and evaluated as described.

### 2.4. Generation of Standard Plasmids

For measuring the absolute gene expression, standard plasmids were generated for α-defensin 5 (DEFA5), lysozyme (Lyz1), wingless and Int 3 (Wnt3), Wnt5a, Wnt9a and matrix metalloproteinase-7 (Mmp7) by using TOPO TA Cloning^®^Kit For Sequencing (lifetechnologies™, Carlsbad, USA). Target genes were amplificated, placed into a plasmid vector by overhanging single-strand ends and transformed into competent One Shot^®^TOP10 + DH5α™-T1^®^ cells (Invitrogen, Carlsbad, CA, USA). The characterization of the plasmid Insert was carried out by sequencing of the plasmid DNA (GATC Biotech AG, Konstanz, Germany).

### 2.5. Organoid Cell Culture

For generating intestinal organoids, crypts from C57BL6J mice were used. Mice were anesthetized using CO_2_ and crypts were isolated from the small intestine by incubation of tissue for 25 min at 4 °C in crypt isolation buffer (CIB, PBSO containing 0.5 M EDTA). Isolated crypts were counted and a total of 500 crypts were plated with 25 µL Matrigel (Corning B.v., Amsterdam, Netherlands) in a 48-well plate. After polymerization of Matrigel, 300 µL of crypt culture medium (CCM) consisting of advanced DMEM/F12 (ThermoFisher Scientific, Karlsruhe, Germany) was supplemented with 100 ng/µL Noggin (PeproTech, East Windsor, NJ, USA), 1 µg/mL R-Spondin (PeproTech, East Windsor, NJ, USA), B-27™ supplement 1× (Invitrogen, Carlsbad, CA, USA), 1 mM N-Acetylcystein (Sigma-Aldrich, Darmstadt, Germany), 0.1 mg/mL Primocin (Invitrogen, Carlsbad, CA, USA) and 50 ng/mL recombinant mouse epidermal growth factor (rm EGF) (Immunotools, Friesoythe, Germany). The resulting organoids were cultured and splitted for a minimum of seven days according to Sato et al. 2009 [33].

### 2.6. Assessment of Cell Viability by Measuring MTT Reduction

To evaluate the effects of vitamin A, vitamin D, signal transducers and activators of transcription 5 (STAT5)-inhibitor STAT5-IN-1, or p38 mitogen-activated protein kinase (p38 MAPK)-inhibitor SB203 580 (5 µM, solved in DMSO; Sigma-Aldrich, Darmstadt, Germany) on organoid cell viability, an MTT reduction assay was performed. For this purpose, organoids were incubated with calcitriol (50 nM, 100 nM, 200 nM, solved in DMSO; Sigma-Aldrich, Darmstadt, Germany), or with retinoic acid (10 µM, 100 µM, 200 µM, solved in DMSO; Sigma-Aldrich, Darmstadt, Germany), or with STAT5-IN-1 (5 µM, 0.5 mM, 1 mM, solved in DMSO; Selleckchem, Planegg, Germany), or with SB203 580 (5 µM, solved in DMSO; Sigma-Aldrich, Darmstadt, Germany), or with a corresponding amount of DMSO as control for 30 h. Then, MTT solution (500 µg/mL; solved in PBSO; Merck, Darmstadt, Germany) was added to organoids to a final concentration of 500 µg/mL. After incubation for 1 h at 37 °C, 5% CO_2_, CCM was discarded and cells were incubated with 20 µL of SDS (for 1 h at 37 °C, 5% CO_2_) to dissolve Matrigel. Finally, 100 µL of DMSO was added (for 1 h at 37 °C, 5% CO_2_) to dissolve reduced MTT, and the optical density was measured at 562 nm on a microplate absorbance reader (BioTek Instruments, Winooski, VT, USA).

### 2.7. Treatment of Organoids

Organoids were treated with calcitriol (50 nM; solved in in DMSO; Sigma-Aldrich, Darmstadt, Germany), or retinoic acid (10 µM, solved in DMSO; Sigma-Aldrich, Darmstadt, Germany), or corresponding amount of DMSO as control for 30 h. Furthermore, cells were incubated with retinoic acid or calcitriol and STAT5-IN-1 (5 µM, solved in DMSO; Selleckchem, Planegg, Germany), or SB203 580 (5 µM, solved in DMSO; Sigma-Aldrich, Darmstadt, Germany) for 30 h. A corresponding amount of DMSO was used as a control.

### 2.8. RNA Isolation and Quantitative Real-Time PCR

For gene expression analysis, total RNA was extracted from tissue using the peqGOLD TriFast system (PEQLAB, Erlangen, Germany). From organoids, total RNA was extracted using the ExtractME Total RNA Kit (blirt S.A.). For real-time PCR (RT-PCR) analysis, a total of 500 ng RNA was reverse-transcribed using the Reverse Transcription System kit and random primers after a DNase digestion step (Promega, Madison, WI, USA). RT-PCR was performed as previously described [21]. The oligonucleotide primer sequences are listed in Table S1. Gene copy numbers of Defa5, Lyz1, regenerating islet-derived protein 3 gamma (Reg3g), cryptdin-1 (Defa1), cryptdin-4 (Defa21), murine β-defensin 1 (Defb1), Defb2, Defb4, Mmp7, peroxisome proliferator-activated receptor gamma (PPARG), nucleotide-binding oligomerization domain-containing protein 2 (NOD2), Wnt3, Wnt5a, Wnt9a, low-density-lipoprotein receptor-related protein 6 (LRP6), T cell-specific transcription factor 1 (Tcf1) and -4 were determined by comparison with a quantitative standard curve generated by serial dilution of plasmid standards and normalized to the copy numbers of mouse housekeeping gene β-actin. Relative gene expression of pan-cryptdin, tumor necrose factor a (Tnfα), myeloid differentiation primary response 88 (Myd88), interleukin 6 (IL6), IL1β, chaperone protein-binding protein (BiP) and activating transcription factor 4 (ATF4) were calculated by comparison to the housekeeping gene β-actin using the ΔΔ-Ct method.

### 2.9. Statistical Analysis

All statistical analyses were performed using GraphPad Prism software 7.0 (GraphPad Software Inc., La Jolla, CA, USA). Normal distribution was tested using the Kolmogorov–Smirnov test. For the statistical comparison of more than two groups, a one-way analysis of variance followed by Dunn’s test or Sidak’s post-test was performed. Differences between two groups were analyzed by using unpaired t-test or Mann–Whitney test. *p*-values of *p* < 0.05 were considered as statistically significant. Correlation analyses were performed with two-tailed Spearman rank correlation, with coefficients in the range of 0.0 to 0.3 (0.0 to −0.3) defined as no correlation, whereas correlations in the range of 0.3 to 0.6 or −0.3 to −0.6 were regarded as positive or negative correlations, respectively.

## 3. Results

### 3.1. Vitamin-Deficient Diets Alter Gut Morphology in Mice

**Figure 1:**
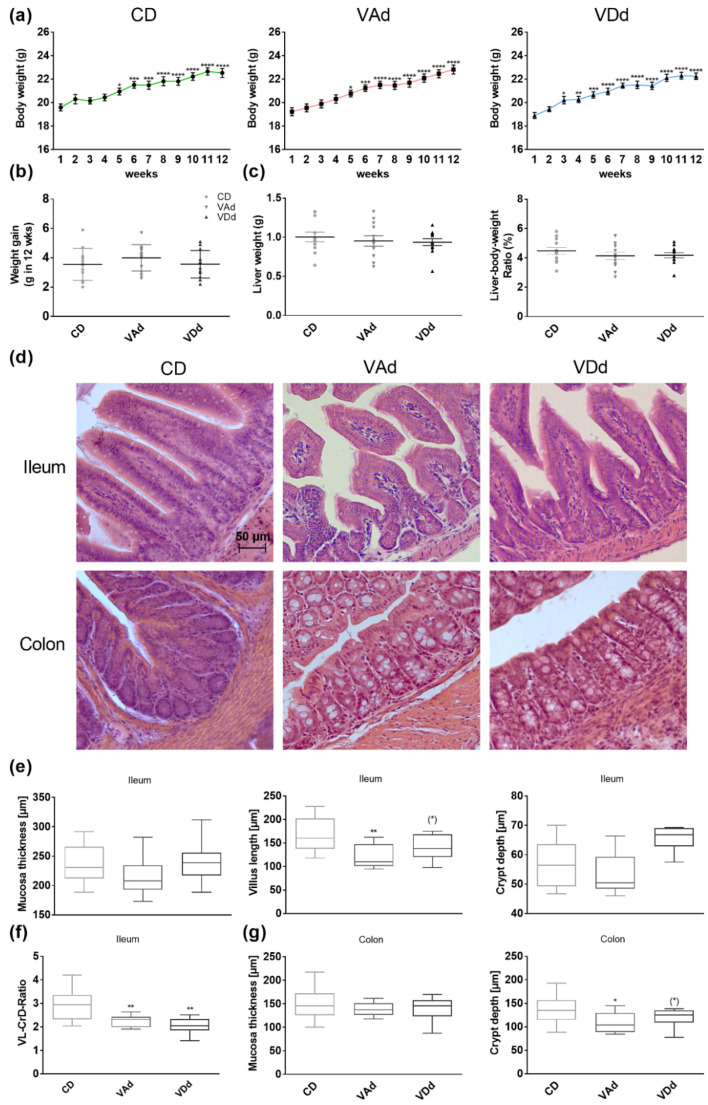
Vitamin-deficient fed mice exhibit morphological changes in the gut. Weight development during 12-week feeding period (**a**). Weight gain in g after 12-week feeding period (**b**) and hepatic markers: liver weight and liver to body weight ratio in percent (**c**) are shown. Representative images of HE staining in ileal and colonic tissue. Scale bar: 50 µm (**d**). Quantification of the mucosa thickness, villus length and crypt depth in ileum (**e**), ileal ratio of villus length to crypt depth (**f**) and of mucosa thickness and depth of the crypts in colon (**g**). Data are presented as means +/− standard error of the mean (*n* = 8–12). Statistical analysis was performed by one-way ANOVA with Sidak´s post-test. Significant differences are indicated: * *p*-value < 0.05; ** *p*-value < 0.01; *** *p*-value < 0.001; **** *p*-value < 0.0001. Abbreviations: CD, control diet; CrD, crypt depth; VAd, vitamin A-free diet; VDd, vitamin D-free diet; VL, villus length.

Overall, 12-week feeding of mice with VAd or VDd did not change weight gain that occurred similarly from week 5 onwards as it did in young mice receiving a CD (*p* < 0.05, Figure 1a–c). There were no differences after completion of 12-week feeding period between the groups, neither in weight gain (Figure 1d) nor in liver parameters, such as liver weight (Figure 1e), liver–body weight ratio (Figure 1f) or liver histology (Figure S1). These data indicate that VAd or VDd do not affect growth, body weight gain or liver weight in female C57BL/6J mice.

Since there is evidence that vitamin deficiency affects gut barrier function, we assessed gut morphology by HE staining and intestinal permeability by FITC and PEG assays. In all, 12-week feeding of VAd, but not VDd, decreased ileal villus length (*p* < 0.01, Figure 1d,e). Analysis of the villus length to crypt depth ratio (VL-CrD-ratio) revealed a decrease when mice were fed a VAd (*p* < 0.01) or a VDd (*p* < 0.01, Figure 1f). Moreover, VAd-fed mice showed reduced colonic crypt depth (*p* < 0.05), which was also decreased in VDd-fed mice by trend (*p* = 0.079, Figure 1d,g). In addition, mRNA analysis of Muc2 gene expression in ileum displayed a reduction by trend in VAd-fed mice (*p* = 0.071, Figure S1). However, these morphological changes were restricted to ileum and colon, while measurements in duodenum and jejunum showed no differences (Figure S1). The determination of plasma DX-400 FITC and PEG in urine samples revealed no significant differences between the different groups (Figure S1).

### 3.2. Vitamin-Deficient Fed Mice Exhibit Reduced Ileal AMP mRNA and Protein Level

**Figure 2:**
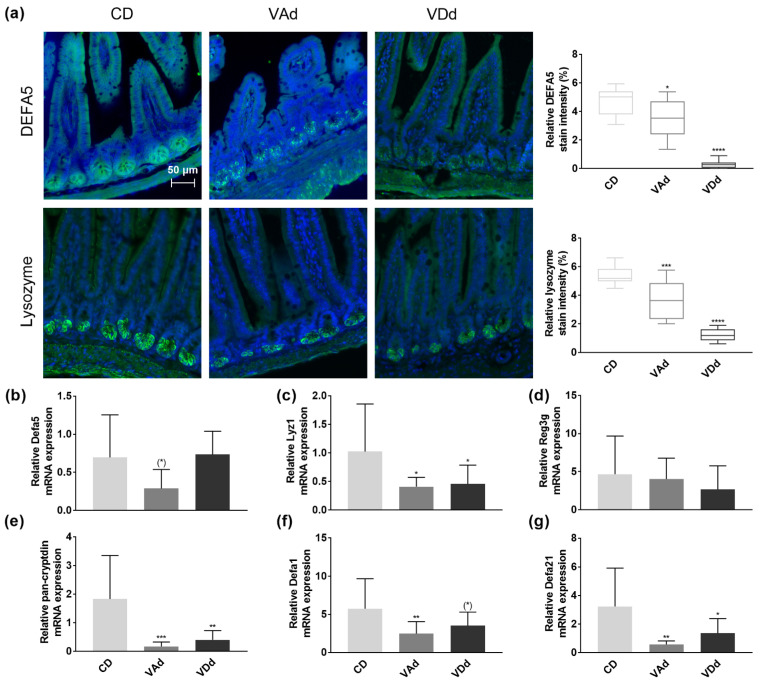
Vitamin A- and D-deficient diets disturb intestinal Paneth cell antimicrobials. Representative images showing DEFA5 or lysozyme expression in green and cell nuclei detected by DAPI in blue. Scale bar: 50 µm. Quantification of relative DEFA5 and lysozyme fluorescence intensity in percent (**a**). mRNA expression levels of Defa5 (**b**), Lyz1 (**c**), Reg3g (**d**), pan-cryptdin (**e**), Defa1 (**f**), Defa21 (**g**) in ileum determined by quantitative RT-PCR. Data are shown as means +/− standard error of the mean (*n* = 8–12). Statistical analysis was performed by one-way ANOVA with Sidak´s post-test. Significant differences are indicated: * *p*-value < 0.05; ** *p*-value < 0.01; *** *p*-value < 0.001; **** *p*-value < 0.0001. Abbreviations: DEFA5, α-defensin 5, Lyz1, lysozyme; Reg3g, regenerating islet-derived protein 3 gamma; Defa1, cryptdin-1; Defa21, cryptdin-4; for other abbreviations, see Figure 1.

Antimicrobial peptides produced and released by Paneth cells are an important defense strategy of the intestinal barrier against pathogens and conducive to maintaining eubiosis. Immunofluorescence analysis in the ileum revealed DEFA5 protein in the granules of Paneth cells located at the base of the crypts when mice were fed a CD, whereas DEFA5 protein levels were reduced in VAd-fed mice (*p* < 0.05) and almost absent in VDd-fed mice (*p* < 0.0001, Figure 1a). In contrast, analysis of Defa5 gene expression showed no difference when mice were fed VDd, and only a minor reduction in the Vad group suggesting post-transcriptional effects (Figure 2b).

Lysozyme, one of the main AMPs in the small intestine, provides two functions, killing bacteria directly by hydrolysis on the one hand, and modulation of bacteria indirectly through the immune system on the other hand [34]. Quantification of lysozyme protein levels in the small intestine of mice revealed that lysozyme was localized in Paneth cells at the base of crypts in CD-fed mice, whereas protein levels were downregulated in VAd (*p* < 0.001) and VDd mice (*p* < 0.0001, Figure 2a). Concordantly, VA- and VD-deficient mice exhibited reduced ileal Lyz1 mRNA expression (*p* < 0.05, Figure 2c).

While VA- and VD-deficient diets had no effect on Reg3g expression (Figure 2d), measurement of total cryptdin by a pan-cryptdin assay demonstrated a reduced expression in VAd- (*p* < 0.001) and VDd-fed mice (*p* < 0.01) compared to control mice (Figure 2e). Consistent with this, ileal mRNA expression of Defa1 was also reduced in VAd-fed mice (*p* < 0.01) and by a trend in mice receiving a VDd (*p* = 0.091, Figure 2f). In addition, vitamin-deficient fed mice exhibited a decrease in ileal Defa21 gene expression (*p* < 0.05, Figure 2g). These data point towards the fact that both VA and VD deficiency deteriorate the antimicrobial peptide defense in the small intestine, resulting in a disturbed intestinal barrier function.

### 3.3. VA- and VD-Deficient Diets Deteriorate Ileal AMP Formation through Wnt, ER Stress and Mmp7

**Figure 3:**
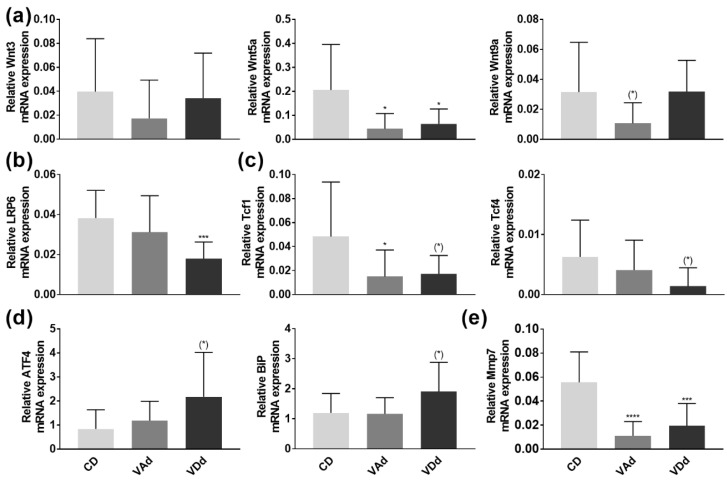
Vitamin-deficient diets disturb AMP formation through Wnt, ER stress and Mmp7. mRNA expression levels of Wnt3, Wnt5a, Wnt9a (**a**), LRP6 (**b**), Tcf1, Tcf4 (**c**) ATF4, BiP (**d**) and Mmp7 (**e**) in the ileum determined by quantitative RT-PCR. Data are shown as means +/− standard error of the mean (*n* = 8–12). Statistical analysis was performed by one-way ANOVA with Sidak´s post-test. Significant differences are indicated: * *p*-value < 0.05; *** *p*-value < 0.001; **** *p*-value < 0.0001. Abbreviations: Wnt, wingless and Int; LRP6, low-density-lipoprotein receptor-related protein 6; Tcf, T cell-specific transcription factor; ATF4, activating transcription factor 4; BiP, chaperone protein-binding protein; Mmp7, peroxisome proliferator-activated receptor gamma; for other abbreviations, see Figure 1.

The wingless-type (Wnt) signaling pathway has been described as an important regulator of intestinal antimicrobial defense [35]. Examination of Wnt signaling molecules Wnt3, Wnt5a, and Wnt9a in the ileum revealed a reduction in Wnt5a expression when mice were fed a VAd or a VDd (*p* < 0.05). Moreover, feeding a VAd reduced Wnt9a mRNA expression by a trend (*p* = 0.098, Figure 3a). Concordantly, we were able to show that gene expression of the LRP6 receptor, transducing Wnt signal into the cell [36], was decreased in mice fed a VDd diet (*p* < 0.0001, Figure 3b). In addition, the expression of the Wnt signaling effector Tcf1 was determined to be reduced in VAd-fed mice (*p* < 0.05) and by trend downregulated in mice receiving a VDd as well (*p* = 0.058). Moreover, VDd-fed mice exhibited a decrease in Tcf4 by trend (*p* = 0.051, Figure 3c).

As endoplasmic reticulum (ER) stress has been identified as a relevant mechanism for impaired AMP protein formation [37], we hypothesized that ileal DEFA5 and lysozyme protein levels were reduced by increased expression of ATF4 and BiP. However, PCR analysis revealed a slight induction in ATF4 (*p* = 0.058) and BiP (*p* = 0.064) in VDd-fed mice, whereas VAd-fed mice showed no differences in gene expression (Figure 3d).

Next, we quantified mRNA expression of Mmp7, which is essential for proteolytic activation of murine α-defensin precursors and which is also located in Paneth cell granules [38,39]. Our data revealed a significant decrease on Mmp7 expression when mice were fed a VAd (*p* < 0.00001) or a VDd (*p* < 0.001, Figure 3e), leading to the conclusion that Paneth cell antimicrobials were not only reduced in gene and protein expression, but also less proteolytic-activated, possibly resulting in an impaired immune defense.

**Figure 4:**
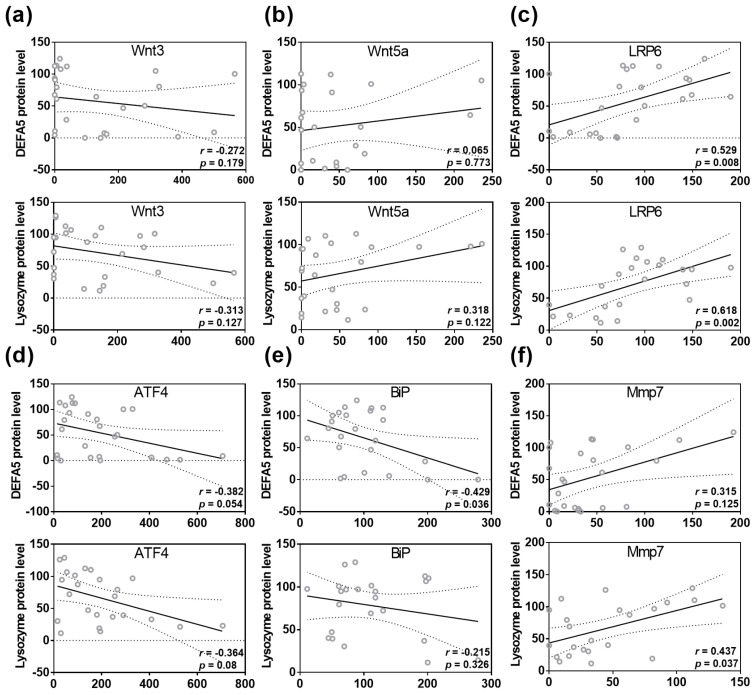
Disturbances in Wnt, ER stress and Mmp7 are correlated with reduced DEFA5 and lysozyme protein levels in the small intestine. Correlation analysis for DEFA5 or lysozyme protein level and Wnt3 (**a**), Wnt5a (**b**), LRP6 (**c**), ATF4 (**d**), BiP (**e**), Mmp7 (**f**) expression. Statistical analysis was performed by two-tailed Spearman rank correlation analysis. Correlations in the range of 0.3 to 0.6 or −0.3 to −0.6 were defined as positive or negative correlations. For abbreviations, see Figure 1, Figure 2 and Figure 3.

Spearman rank correlation analysis identified a correlation between ileal lysozyme protein levels and the expression of the Wnt signaling molecules Wnt3 (r = −0.313; *p* = 0.127) and Wnt5a (r = 0.318; *p* = 0.122, Figure 4a,b). Moreover, we found a strong positive correlation between ileal DEFA5 protein quantity and LRP6 expression (r = 0.529, *p* = 0.008), as well as between ileal lysozyme fluorescence signal and LRP6 (r = 0.618, *p* = 0.002, Figure 4c). However, no correlation was found between lysozyme or DEFA5 protein levels and Wnt9a, Tcf1, and Tcf4 gene expression (Figure S2). Furthermore, there was a negative correlation between DEFA5 protein levels and ATF4 (r = −0.382; *p* = 0.054) and BiP (r = −0.429; *p* = 0.036), whereas lysozyme protein levels were negatively correlated with ATF4 gene expression (r = −0.364; *p* = 0.08, Figure 4d,e). Additionally, Spearman rank correlation analysis revealed a positive correlation between DEFA5 fluorescence signal and the activating enzyme Mmp7 (r = 0.315; *p* = 0.125) and a strong correlation between lysozyme protein levels and Mmp7 gene expression in the ileum (r = 0.437; *p* = 0.037, Figure 4f).

Collectively, these results indicate that VA and VD deficient diets impaired Wnt signaling pathway, increased ER stress, and decreased Mmp7 expression, which in turn reduce ileal AMP levels and proteolytic activation in the small intestine.

### 3.4. VA and VD Induce AMP Gene Expression In Vitro through STAT5 Signaling Pathway

There is evidence that VA and VD regulate gene expression through the MAPK [40,41] and Janus kinase (Jak)/STAT signaling pathway [42]. Based on our previous results, we therefore hypothesized that the p38 MAPK and STAT5 signaling pathway might be involved in VA- and VD-mediated effects. To evaluate this, we used the organoid cell model.

**Figure 5:**
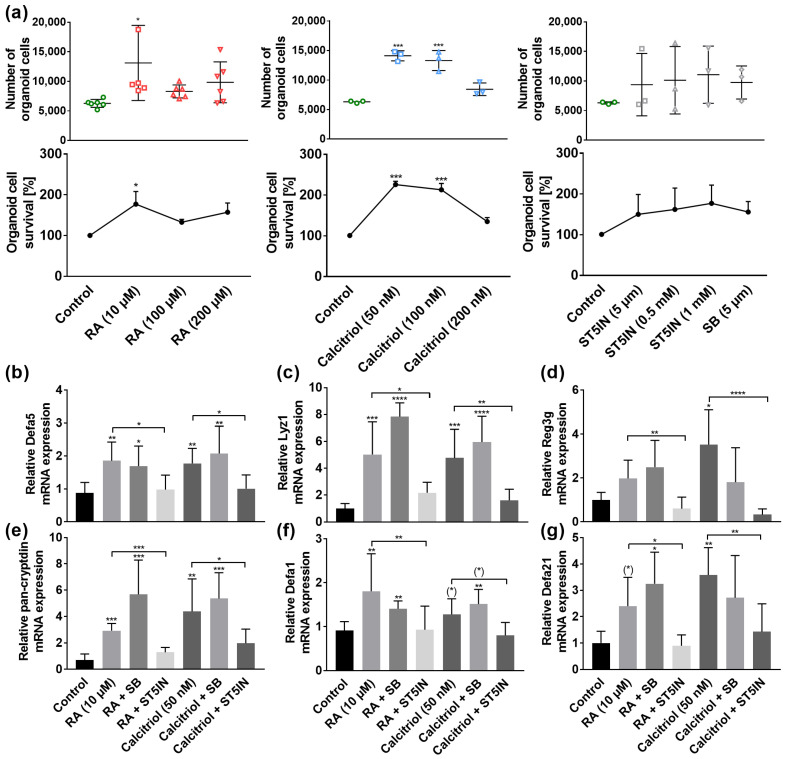
Retinoic acid (RA) and calcitriol regulate AMPs in murine organoids in vitro through STAT5 signaling pathway. For MTT assay, organoids were treated with RA (10 µM, 100 µM, 200 µM), calcitriol (50 nM, 100 nM, 200 nM), ST5IN (5 µM, 0.5 mM, 1 mM) or with SB (5 µM) for 30 h. The total number of organoids and cell survival by quantification of fluorescence signals are shown (*n* = 3–6) (**a**). Organoids were treated with RA (10 µM) or calcitriol (50 nM) in combination with ST5IN (5 µM) or SB (5 μM) for 30 h. Relative mRNA expression level of Defa5 (**b**), Lyz1 (**c**), Reg3g (**d**), pan-cryptdin (**e**), Defa1 (**f**) and Defa21 (**g**) determined by quantitative RT-PCR derived in organoids from the small intestine of healthy C57BL/6 mice (*n* = 3–6). Data are presented as means ± SEM and were analyzed by one-way ANOVA with Sidak´s post-test or unpaired *t*-test. Significant differences are indicated: * *p*-value < 0.05; ** *p*-value < 0.01; *** *p*-value < 0.001; **** *p*-value < 0.0001. Abbreviations: RA, retinoic acid; SB, p38 MAPK inhibitor SB203 580; ST5IN, STAT5 inhibitor STAT5-IN-1; for other abbreviations, see Figure 1 and Figure 2.

To exclude toxicological effects, we first examined the effects of retinoic acid (RA), calcitriol, p38 inhibitor SB203 580 (SB) and STAT5 inhibitor STAT5-IN-1 (ST5IN) on cell viability by an MTT reduction assay. Quantification of organoid cell number and cell survival revealed that 10 µM RA enhanced cell number and viability (*p* < 0.05, Figure 5a). Similarly, 50 nM and 100 nM calcitriol increased cell number and viability (*p* < 0.001), whereas 200 nM calcitriol had no effects (Figure 5a). Moreover, treatment with neither STAT5-IN-1 nor SB203 580 had any effect on organoid cell number and cell survival (Figure 5a).

Stimulating organoids with 10 µM RA resulted in an induction in Defa5 (*p* < 0.01), Lyz1 (*p* < 0.001), pan-cryptdin (*p* < 0.001), Defa1 (*p* < 0.01), and Defa21 gene expression by a trend (*p* = 0.0776). Concordantly, 50 nM of calcitriol induced Defa5 (*p* < 0.01), Lyz1 (*p* < 0.001), Reg3g (*p* < 0.05), pan-cryptdin (*p* < 0.01), Defa1 by a trend (*p* = 0.0745), and Defa21 (*p* < 0.01, Figure 5b–g). Moreover, our results revealed that these effects of RA and calcitriol were absent when STAT5 signaling pathway was blocked (Figure 5b–g). In contrast, stimulation of organoid cells with vitamins and p38 inhibitor SB resulted in a comparable induction in AMP gene expression as with RA or calcitriol only. These results indicate that VA and VD induce AMP mRNA expression through the Jak/STAT5 signaling pathway, whereas MAPK pathway is not involved in the VA- and VD-related gene regulation.

### 3.5. VA- and VD-Deficient Diets Increase Colonic β-Defensin Expression and Inflammation

**Figure 6:**
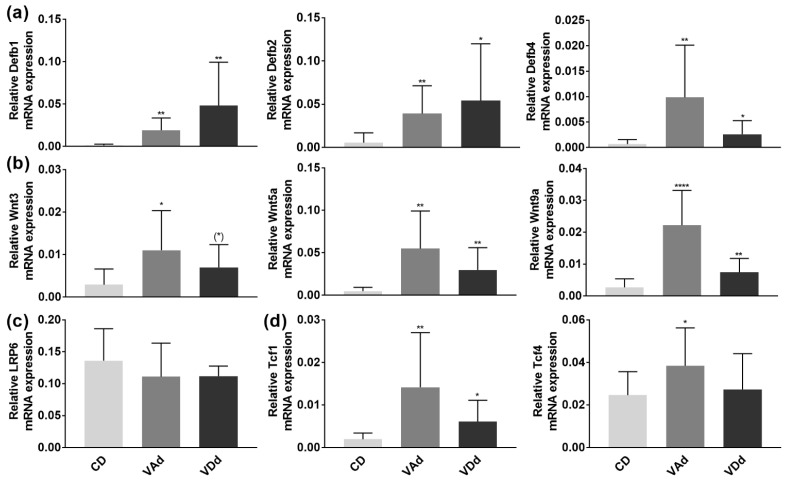
Vitamin-deficient diets enhance β-defensins in the colon through increased Wnt signaling pathway. Absolute mRNA expression levels of Defb1, −2, −4 (**a**), Wnt3, -5a, -9a (**b**), LRP6 (**c**) Tcf1 and Tcf4 (**d**) in the colon determined by quantitative RT-PCR. Data are shown as means +/− standard error of the mean (*n* = 8–12). Statistical analysis was performed by one-way ANOVA with Sidak´s post-test. Significant differences are indicated: * *p*-value < 0.05; ** *p*-value < 0.01; **** *p*-value < 0.0001. Abbreviations: Defb, murine β-defensin; for other abbreviations, see Figure 1 and Figure 3.

To maintain the microbial homeostasis in the colon, epithelial cells build a chemomechanical barrier, including the formation and release of the constitutively expressed β-defensin Defb1. In case of inflammation or infection, the synthesis of additional defensins, such as Defb2-4 and other antimicrobial peptides, is stimulated [43]. PCR analysis revealed a significant induction in Defb1 (*p* < 0.01), Defb2 (*p* < 0.05), and Defb4 (*p* < 0.05) gene expression (Figure 6a) when mice were fed a VAd or VDd for 12 weeks. Furthermore, our data point towards the fact that increased β-defensin expression resulted from augmented Wnt signaling pathway, as VAd mice showed an induction in Wnt3 (*p* < 0.05), Wnt5a (*p* < 0.01), and Wnt9a (*p* < 0.0001). Similarly, VDd-fed mice exhibited an increment in Wnt3 gene expression by trend (*p* = 0.061), as well as an increase in Wnt5a (*p* < 0.01) and Wnt9a (*p* < 0.01) mRNA expression (Figure 6b). While LRP6 expression remained unchanged (Figure 6c), VAd- and VDd-fed mice showed an induction in Tcf1 (*p* < 0.05) and Tcf4 (*p* < 0.05) expression (Figure 6d).

Concordant to the β-defensin expression, the assessment of inflammatory markers revealed a trend of an increased PPARG mRNA expression (*p* = 0.082), as well as an induction in NOD2 (*p* < 0.01) and Myd88 (*p* < 0.05) gene expression in VAd mice. In comparison, VDd-fed mice exhibited an increased NOD2 expression by trend (*p* = 0.065) and an induction in Myd88 mRNA expression (*p* < 0.05; Table 2).

Moreover, VAd-fed mice exhibited increased mRNA expression levels of the proinflammatory cytokines Tnfα (*p* < 0.01), IL6 (*p* < 0.01), and IL1β (*p* < 0.05) in the colon. In addition, VDd mice had enhanced mRNA expression level of Tnfα (*p* < 0.001) and IL-6 (*p* < 0.05, Table 2).

Our data suggest that VA- and VD-deficient diets led to increased colonic inflammation and increased β-defensin responses, which were regulated by the Wnt signaling pathway.

## 4. Discussion

In the present study, we were able to show that VA and VD regulate the intestinal barrier function, especially by modulating antimicrobial peptide defense. Using a mouse model, we demonstrated that both VA- and a VD-deficient diets resulted in an impaired antimicrobial peptide expression and proteolytic activation. Moreover, our data point towards the fact that VA- and VD-mediated effects on AMP formation were dependent on the Wnt and STAT5 signaling pathways.

Our results revealed that from week 5 onwards, all mice significantly gained body weight and that neither VAd nor VDd affected body weight change or hepatic markers such as liver weight or liver–body weight ratio. These findings complement existing research showing that VDd has no effects on body weight in 6-week-old C57BL/6 mice receiving a diet containing less than 25 IU vitamin D3 for 6 weeks [44]. Similarly, VA deficiency has been shown to affect weight gain or liver weight [45]. However, it is well known that especially VA plays a critical role in embryonic development [46,47], as it is necessary for early cell proliferation and differentiation [48]. Studies revealed that VA deficiency resulted in reduced weight development and growth retardation in 5-week-old rats [49,50], whereas high doses of all-trans RA decreased body weight gain and bone development in growing rats [51]. Concordantly, 3-week-old male mice receiving VDd displayed lower weight gain than VD-sufficient mice, although energy intake was not changed between the groups [52]. Moreover, 5-week feeding of VDd in young female C57BL/6 mice resulted in reduced weight gain [53], confirming the role of age and VA- or VD-deficient nutrition on the body weight.

We provide evidence that feeding a VAd or VDd decreased VL and VL–CrD ratio in the ileum and CrD in the colon, whereas mucosa thickness and gut permeability measured by PEG and FITC marker tests remained unchanged. VL and the VL–CrD ratio are indicators of intestinal health, as they are associated with improved nutrient uptake [54]. Furthermore, CrD reflects the development of crypt cells, whereby deeper crypts have higher secretion capacity [55]. The results of our study are in accordance with current findings, as VD supplementation improved epithelial proliferation, enterocyte apoptosis rate and IEC turnover in the small intestine and colon in cholestasis rats [56]. In addition, crypt stem cells exhibited high VDR expression, which was absent in VDR-KO mice resulting in reduced stem cell proliferation and crypt depth [57]. Consistently, Yeung et al. [52] showed that VDd led to crypt hyperplasia, loss of epithelial integrity as well as a decreased mucosa thickness in colon of mice. VA was determined to improve mucosal damage and immunity during intestinal Salmonella infections in rats, whereas VA deficiency increased the number of small intestinal dendritic cells [55]. Moreover, VAd-fed rats showed increased number of goblet cells in the small intestine and colon [58] and VAd and VDd changed mucin dynamics by reducing Muc2 and Muc3 mRNA expression in ileum and colon [58,59]. Although our results revealed no effects of VAd or VDd on intestinal permeability, there is evidence that VD supplementation improved intestinal permeability and bacterial translocation in mice fed a high-fat diet and liver cirrhosis [56,60], whereas VA supplementation seems not to effect gut permeability [61].

Moreover, we were able to demonstrate that RA and calcitriol regulate α-defensin expression ex vivo in organoid cells and that 12-week feeding of a VAd or VDd reduced ileal Defa5 and Lyz1 protein and mRNA expression, as well as gene expression of cryptdins in mice. Concordantly, Amit-Romach et al. [58] demonstrated that VAd reduced Defa6 gene expression in jejunum, ileum, and colon of rats, which has been associated with increased penetration of bacteria into host’s cellular surface and bacterial overgrowth. Similarly, mice fed a high-fat and VD-deficient diet exhibited reduced ileal Defa5, Defa1 and Defb1 mRNA expression, which was restored by VD supplementation [59]. Lu et al. [18] showed that decreased VDR signaling correlated with reduced Lyz1 gene expression in patients with ulcerative colitis, which was associated with impaired anti-bacterial abilities and inflammatory responses. Furthermore, reduced serum 25-hydroxy vitamin D levels were associated with decreased ileal Defa2 and Defa5 expression in mice. Additionally, ileal expression of Mmp7 and Lyz1 markers for Paneth cell functionality were reduced [62]. This is in line with our findings that antimicrobial activation by proteolytic cleavage of Mmp7 was reduced in mice fed a VDd or VAd. Additionally, retinoic acid has been shown to regulate Mmp7 homeostasis in lungs of asthma mouse model and healthy lungs in humans [63,64], as well as in human colon cancer and cancer cell lines [65,66].

Our study showed that VA and VD deficiency impairs Paneth cell antimicrobial function in mice and highlighted the role of the Wnt signaling pathway in mediating the effects of VA and VD on α-defensins. Consistent with our results, there is evidence that both VA and VD regulate the Wnt signaling pathway. Accordingly, Gröschel et al. [67] showed that VD reduced β-catenin and Wnt signaling pathways in adenoma cells, regulating cell differentiation. Moreover, VDd disturbed Wnt signaling pathway in mice, such as decreased TCF4 expression. Furthermore, VD has been shown to induce the Wnt antagonist DICKKOPF-1, inhibiting the formation of β-catenin–TCF complexes. Additionally, VD is a direct antagonist of Wnt-β-catenin signaling by promoting binding of VDR to β-catenin [68]. Similarly, Wnt and RA signaling pathways have been associated with the development of human colorectal carcinoma with adenomatous polyposis coli (APC) mutations, as the upregulation of Wnt signaling by downregulated RA signaling resulted in immature APC-mutant colon tissue [69].

Using organoid cell culture, we demonstrated that RA- and VD-mediated regulation of α-defensins was almost absent by blocking STAT5 signaling pathway. Consistently, all-trans retinoic acid (ATRA) has been demonstrated to induce the expression of E74-like factor 5 of STAT5β, as well as the phosphorylation of STAT5β in mammary epithelial cell line, thereby regulating fatty acid synthesis [70]. Additionally, STAT5 binding sites were determined to overlap with retinoic acid receptor (RAR) responsive element regulating the transcription of target gene [71]. Similar findings have been described for VD. In human monocytes, VD treatment promoted the formation of the VD/VDR complex and increased the accessibility of binding sites. Moreover, these binding sites were attached by p-STAT5, resulting in increased VDR/STAT5 complexes, which was associated with an induction in anti-inflammatory cytokines [72,73]. While our results could not reveal effects of the p38 MAPK signaling pathway, other studies indicated that both VD and VA can modulate gene expression through regulation of p-38-MAPK and p38/Erk signaling [74,75].

In the present study, we provide evidence that VAd and VDd increased colonic inflammatory processes, as the expression of β-defensins, Wnt signaling molecules, and inflammatory markers was induced. In accordance with our results, β-defensins have been demonstrated to be induced by bacteria [76,77] and bacterial substances [78]. Furthermore, inflammatory stimuli such as Tnfα and IL1β were involved in the regulation of Defb2 expression [79] and mice exhibiting reduced VD signaling had increased intestinal inflammation and dysbiosis [80,81]. This is in line with our results revealing increased colonic expression of Tnfα, Myd88, IL6, and IL1β in VAd- and VDd-fed mice. Elevated Tnfα levels were also associated with intestinal barrier dysfunction, TJ disruption and disturbed epithelial morphology [82]. Wang et al. [56] showed increased intestinal Tnfα levels in cirrhotic rats, resulting in intestinal crypt cell hyperplasia and dysregulated intestinal epithelial turnover. In addition, the expression of junctional proteins was impaired by Tnfα in vitro [83].

Finally, we were able to detect enhanced Wnt signaling pathway activity in the colon, as Wnt3, Wnt5a, and Wnt9a were increased in VAd- and VDd-fed mice. Besides regulating AMP expression, the Wnt signaling pathway modulates intestinal barrier function by inducing cell differentiation and proliferation [84]. While inhibition of the Wnt signaling pathway has been associated with reduced proliferation of stem cells [84], enhanced Wnt pathway activation has been identified to be involved in tumorigenesis [85]. Zhang et al. [86] demonstrated that the Wnt/β-Catenin signaling pathway was promoted by Ribonuclease H1 antisense RNA 1 in lung cancer tissue and in non-small cell lung cancer (NSCLC) cell lines. In addition, PT130 cells and tumor organoid culture studies assumed that activation of dynamin-related protein 1 (Drp1) promotes fatty acid-induced metabolic reprogramming to enhance Wnt signaling in colon cancer [87]. Recently, Sana et al. [88] were able to show that vitamin deficiency as well as the expression of VDR and retinoid X receptor (RXR) correlated with blood cancer diagnosis in patients.

## 5. Conclusions

In conclusion, our data demonstrated that vitamin A- or D-deficient diet plays a critical role in the regulation of gastrointestinal morphology and antimicrobial peptide defense in the small intestine. Furthermore, we were able to demonstrate that vitamins A and D regulate antimicrobial peptides through the Wnt signaling pathway, Mmp7, and Jak/STAT signaling. Moreover, vitamin A and D deficiency increased inflammatory processes in the colon. The study of the molecular mechanisms by which vitamins modulate the antimicrobial gut barrier function provides new insights into the regulation of the intestinal barrier and offers novel approaches for dietary strategies aiming to improve host defense against infections and to ameliorate gut barrier dysfunction.

## Figures and Tables

**Table 1:** Nutrient formulation of experimental diets fed to female C57BL/6J mice for 12 weeks (n = 12 per group).

	CD, Ssniff ^®^ EF R/M Control	Ssniff^®^ EF R/M Vitamin-A-Free Vitamin A < 120 IE	Ssniff^®^ EF R/M Vitamin-D-Free Vitamin D < 5 IE
Diet specification	CD	VAd	VDd
ME [MJ/kg]	18.0	18.0	18.0
Carbohydrates [kJ%]	61	61	61
Mono- and Disaccharides [%]	10.8	10.8	12.9
Protein [kJ%]	30	30	30
Fat [kJ%]	9	9	9
Vitamin A [IE]	15,000	<120	15,000
Vitamin D [IE]	1500	1500	<5
Minerals [%]	2.9	2.9	1.63
Sodium [%]	0.19	0.19	0.19
Starch [%]	46.8	46.8	46.8
Crude ash [%]	5.6	5.6	4.0
Crude fiber [%]	5.0	5.0	5.0
Crude protein [%]	20.8	20.8	20.8
Crude fat [%]	4.2	4.2	4.2

Abbreviations: ME, metabolizable energy; CD, control diet; VAd, vitamin A-free diet; VDd, vitamin D-free diet.

**Table 2:** Inflammatory markers measured in the colon tissue.

Treatment	PPARG	NOD2	Tnfα	Myd88	IL6	IL1β
CD	0.086 ± 0.011	0.003 ± 0.0001	1.14 ± 0.3	3.1 ± 1.3	0.61 ± 0.34	0.8 ± 0.22
VAd	0.124 ± 0.18 *	0.015 ± 0.003 **	7.04 ± 1.73 **	16.64 ± 6.22 *	15.77 ± 4.7 **	8.61 ± 2.91 *
VDd	0.083 ± 0.017	0.01 ± 0.003 *	8.11 ± 1.68 ***	27.54 ± 10.02 *	8.28 ± 3.5 *	3.14 ± 1.83

Vitamin-deficient diets increase colonic inflammation. mRNA expression of PPARG, NOD2, Tnfα, Myd88, IL6, IL1β in the colon determined by quantitative RT-PCR. Data are shown as means +/− standard error of the mean (*n* = 8–12). Statistical analysis was performed by one-way ANOVA with Sidak´s post-test. Statistics: * indicates differences relative to CD. * *p*-value < 0.05; ** *p*-value < 0.01; *** *p*-value < 0.001. Abbreviations: PPARG, peroxisome proliferator-activated receptor gamma; NOD2, nucleotide-binding oligomerization domain-containing protein 2; Tnfα, tumor necrose factor alpha; Myd88, myeloid differentiation primary response 88; IL, interleukin; for other abbreviations, see Figure 1.

## Data Availability

The data presented in this study are available upon justified request to the corresponding author.

## Supplementary Materials

The following supporting information can be downloaded at: https://www.mdpi.com/article/10.3390/nu15020376/s1, Table S1: Primers used in quantitative real-time PCR; Figure S1: HE staining in hepatic, duodenal and jejunal tissue, Intestinal permeability by Dx-4000-FITC in the portal venous plasma and by PEG4000 recovery in the urine; Figure S2: Correlation analysis for DEFA5 or lysozyme protein level and Wnt9a, Tcf1, and Tcf4 expression in the small intestine.
