# Supplementary material for: Vitamin A- and D-Deficient Diets Disrupt Intestinal Antimicrobial Peptide Defense Involving Wnt and STAT5 Signaling Pathways in Mice
Source: Nutrients. 2023 Jan 11;15(2):376. doi: 10.3390/nu15020376 (PMC9863741; doi:10.3390/nu15020376)
Supplement: Supplementary file 1 [file nutrients-15-00376-s001.zip › nutrients-2094969-supplementary.pdf]

Supplements

Table S1. Primers used in quantitative real-time PCR.

Primer	Forward (5′-3′)	Reverse (5′-3′)
Actb	GCTGAGAGGGAAATCGTGCGTG	CCAGGGAGGAAGAGGATGCGG
Pan-cryptdin	AAGAGACTAAAACTGAGGAGCAGC	GGTGATCATCAGACCCCAGCATCAGT
Defa1	TCAAGAGGCTGCAAAGGAAGAGAAC	TGGTCTCCATGTTCAGCGACAGC
Defa21	CCAGGGGAAGATGACCAGGCT	TGCAGCGACGATTTCTACAAAGGC
PPARG	TTGCTGAACGTGAAGCCCATCGAGG	GACGAGGTCCTCTAGATGTTCCTG
Lyz1	GCCAAGGTCTACAATCGTTGTGAGTTG	CAGTCAGCCAGCTTGACACCACG
Reg3g	TTCCTGTCCTCCATGATCAAAA	CATCCACCTCTGTTGGGTTCA
Defb1	TCCAATAACATGCATGACCA	TCATGGAGGAGCAAATTCTG
Defb2	TCAGAGCCATTTGTCCTCCT	TGACTTCCATGTGCTTCCTTC
Defb4	TCCAATAACATGCATGACCA	TCATGGAGGAGCAAATTCTG
Defa5	TCAAAAAAGCTGATATGCTATTG	AGCTGCAGCAGAATACGAAAG
Mmp7	TTCAAGAGGGTTAGTTGGGGGACTG	CCGCCTCTACGAGTGAAACTGTT
Myd88	CAAAAGTGGGGTGCCTTTGC	AAATCCACAGTGCCCCCAGA
Tnfα	ACCACCATCAAGGACTCA	AGGTCTGAAGGTAGGAAG
NOD2	GGCACCTGAAGTTGACATTTTGC	ATCTCCCACAGAGTTGTAATCC
IL6	AGTCACAGAAGGAGTGGCTA	CTGACCACAGTGAGGAATGT
IL1β	ACGGATTCCATGGTGAAGTC	GAGTGTGGATCCCAAGCAAT
BiP	TCATCGGACGCACTTGGAA	TGGTTTGCCCACCTCCAATA
ATF4	GGAAGACACTCCCTCTGACAATG	ACGGGAACCACCTGGAGAAG
Muc2	GATGGCACCTACCTCGTTGT	GTCCTGGCACTTGTTGGAAT
Wnt3	TGGAACTGTACCACCATAGATGAC	GTAGCGGAGCCGACCACA
Wnt5a	ATGGTGTGTCTGGCTCCTG	CAGTGGCTTCATTGGTAGTGCT
Wnt9a	GCAGGACTTTCTCAAGGACA	CCCTGCCAAAGACAGAAGTA
LRP6	TGGTGGCTTGTGGTGCTG	CAATGGCGATGCGAACTG
Tcf1	CCAGGACTCACCCTCGTA	TTCTGTGCCTAGCAACCAA
Tcf4	CTGGTCTGCACGGGATAA	TGTGACTTGGCGTCTTGG

Abbreviations: Actb, β-Actin; pan-cryptdin, pancryptdin; Defa1, cryptdin-1; Defa5, α-defensin 5; Defa21, cryptdin-4; PPARG, peroxisome proliferator activated receptor gamma; Lyz1, lysozyme; Reg3g, regenerating islet-derived protein 3 gamma; Defb, murine β-defensin; Mmp7, matrix metalloproteinase-7; Myd88, myeloid differentiation primary response 88; Tnfα, tumor necrose factor a; NOD2, nucleotide-binding oligomerization domain-containing protein 2; IL, interleukin; BiP, chaperone protein-binding protein; ATF4, activating transcription factor 4; Muc, Mucin; Wnt, wingless and Int; LRP6, low-density-lipoprotein receptor related protein 6; Tcf, T cell-specific transcription factor.

Figure S1:

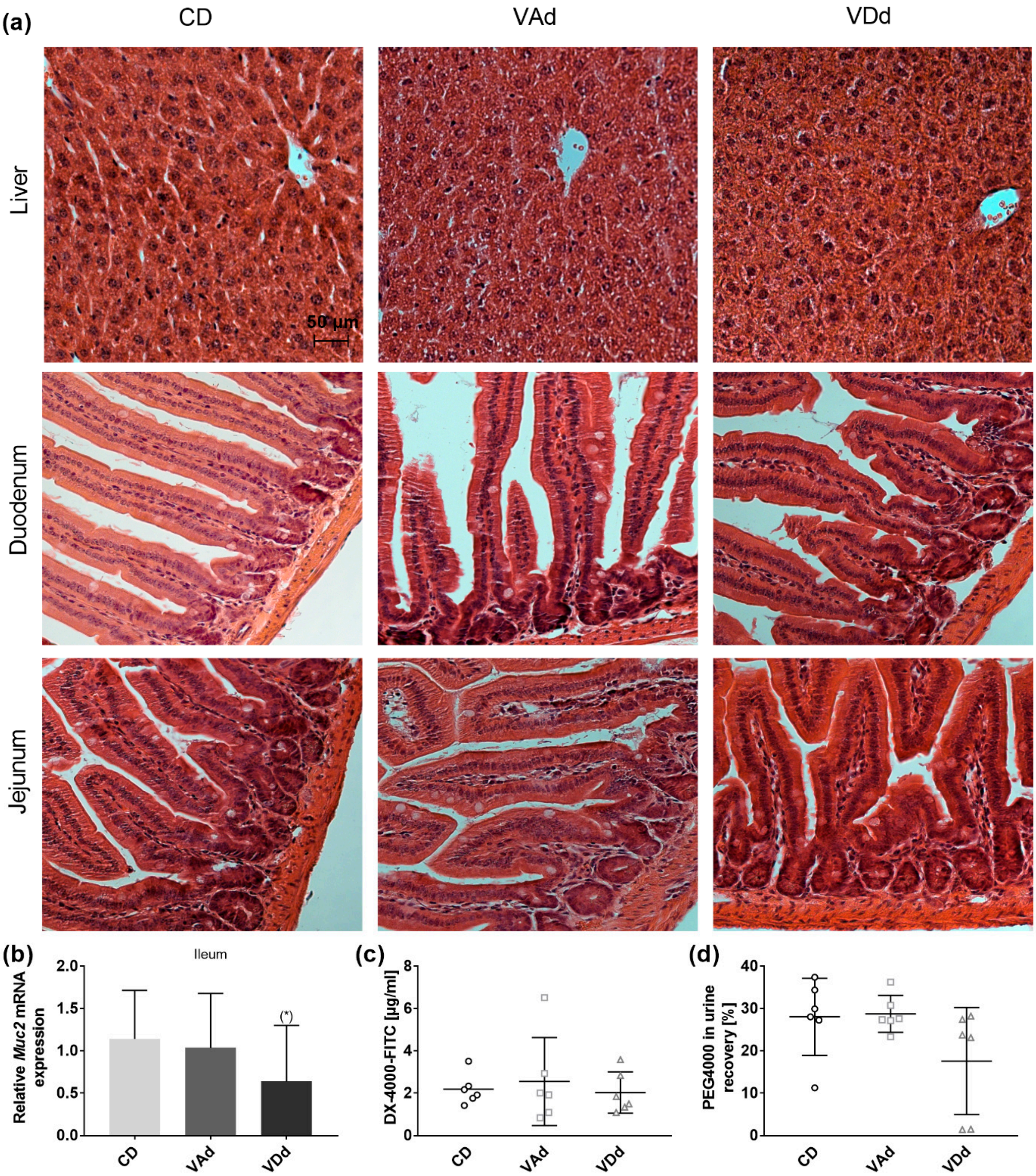

Figure S1. Representative images of HE staining in hepatic, duodenal and jujenal tissue. Scale bar: 50 µm (a). Relative mRNA expression of Muc 2 in the ileum quantified by RT-PCR (b). Intestinal permeability was determined by Dx-4000-FITC in the portal venous plasma (c) and by PEG4000 recovery in the urine (d) Data are presented as means +/- standard error of the mean (SEM) (n = 8-12). Statistical analysis was performed by one-way ANOVA with Sidak´s post-test. Abbreviations: CD, control diet; VAd, vitamin A free diet; VDd, vitamin D free diet, DX-4000-FITC, fluorescein isothiocyanate-dextran; Muc2, mucin 2; PEG, polyethylene glycol.

Figure S2:

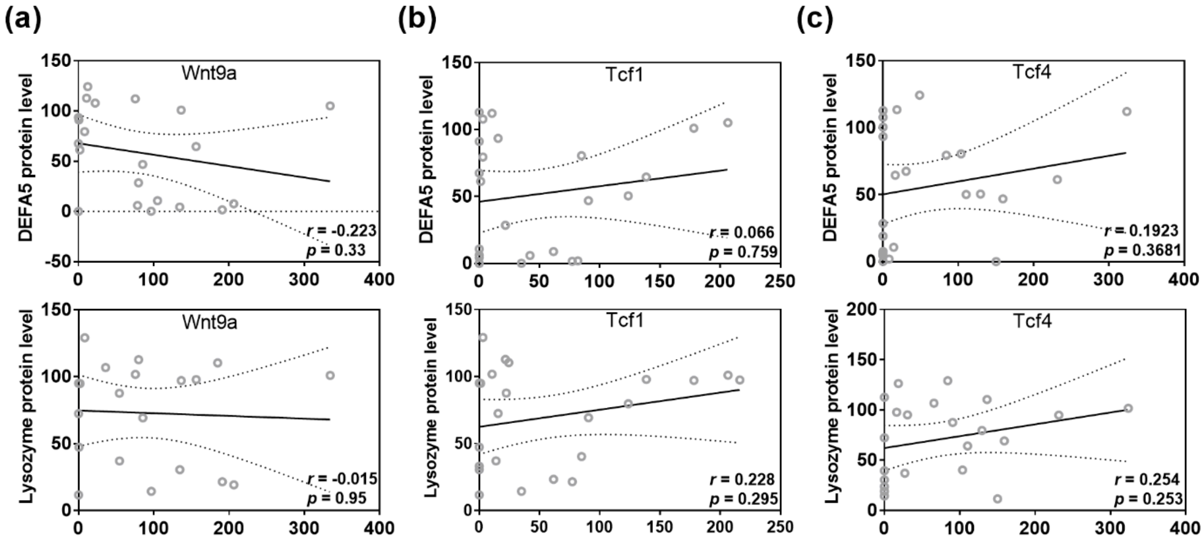

Figure S2. Correlation analysis for DEFA5 or lysozyme protein level and Wnt9a (a), Tcf1 (b), and Tcf4 (c) expression. Statistical analysis was performed by two-tailed Spearman-rank correlation analysis. Correlations in the range of 0.3 to 0.6 or −0.3 to −0.6 were defined as positive or negative correlations. For abbreviations, see Figure 2, and 3.
